# One in ten ever-married women who visited health facilities for various reasons have pelvic organ prolapse in Harari regional state, Eastern Ethiopia

**DOI:** 10.1186/s12905-022-01817-8

**Published:** 2022-06-11

**Authors:** Dawit Abebe, Mohammed Abdurke Kure, Enku Afework Demssie, Sinetibeb Mesfin, Melake Demena, Merga Dheresa

**Affiliations:** 1 School of Nursing and Midwifery, College of Medicine and Health Sciences, Jigjiga University, Jigjiga, Ethiopia; 2 School of Nursing and Midwifery, College of Health and Medical Sciences, Haramaya University, Harar, Ethiopia; 3 Department of Public Health, Adama General Hospital and Medical College, Adama, Ethiopia; 4 School of Public Health, College of Health and Medical Sciences, Haramaya University, Harar, Ethiopia

**Keywords:** Pelvic organ prolapse, Associated factors, Risk factors, Eastern Ethiopia

## Abstract

**Background:**

Pelvic organ prolapse remains a neglected public health problem in developing countries. The burden of pelvic organ prolapse varies by region and ranges from 9 to 20%. It poses an impact on women’s quality of life and affects their role at the community and family level. Although it has negative consequences and extensive burden, the true feature of pelvic organ prolapse is not well known among ever-married women attending health facilities for various reasons in the study area. Therefore, this study was aimed to assess the magnitude of pelvic organ prolapse and associated factors among ever-married women attending health care services in public Hospitals, Eastern Ethiopia.

**Methods:**

A facility-based cross-sectional study design was conducted from March 4th to April 5th, 2020 among 458 ever-married women attending public Hospitals in Harar town, Eastern Ethiopia. The study subjects were selected through systematic sampling. The data were collected using a structured questionnaire through face-to-face interviews. Data were analyzed using SPSS version 22 (IBM SPSS Statistics, 2013). The prevalence was reported by proportion and summary measures. Predictors were assessed using a multivariable logistic regression analysis model and reported using an adjusted odds ratio with 95% CI. Statistical significance was declared at p-value < 0.05.

**Result:**

Of 458 women enrolled in the study, 10.5% of them had pelvic organ prolapse based on women’s reporting of symptoms. History of lifting heavy objects [AOR = 3.22, 95% CI (1.56, 6.67)], history of chronic cough [AOR = 2.51, 95% CI (1.18, 5.31)], maternal age of greater than or equal to 55 years [AOR = 3.51, 95% CI (1.04, 11.76)], history chronic constipation (AOR = 3.77, 95% CI (1.54, 9.22) and no history of contraceptive utilization [AOR = 2.41, 95% CI (1.13, 5.05)] were significantly associated with pelvic organ prolapse.

**Conclusion:**

In this study, one in ten ever-married women who visited health facilities for various reasons have pelvic organ prolapse. Modifiable and non-modifiable risk factors were identified. This result provides a clue to give due consideration to primary and secondary prevention through various techniques.

## Background

Pelvic organ prolapse (POP), or genital prolapse, is the descent from or through the vaginal opening of one or more pelvic structures from the normal anatomy. The pelvic structure includes the vagina, uterus, bladder, urethra, and rectum [1, 2]. POP remains a public health challenge in the developing world [3]. POP affects around 9% of women worldwide [4]. The prevalence of POP varies by region and methods of assessment. The prevalence ranges from 12 to 65% [5, 6]. Multiparity [2, 7], advanced maternal age [8], prolonged labor [9, 10], lifting of heavy objects [6, 11], and obesity [12] are identified as risk factors for POP.

In developing countries like Ethiopia, the situation is far worse [13, 14], especially in a setting where a high fertility rate of more than 4.6 is reported [15]. In addition, the trends with early marriage soon after followed by early childbearing that leads to many vaginal deliveries for her lifetime and home delivery contributed to more than 80% of the problem [16] and frequent heavy lifting related to the socio-economic role of women make the problem unbearable [12]. The lifetime risk of surgery for POP in the general female population is about 19–20% [17, 18]. In addition, POP become one of the three major indications for hysterectomy, which account for about 23% [19]. In Ethiopia, one in five women suffers from pelvic floor disorder, of which 9.5% is attributed to POP [20]. A recent study indicated a national estimated prevalence of pelvic organ prolapse in Ethiopia is 23.52% and about 15–40.7% of major gynecologic operations are due to POP [21–23].

Furthermore, POP has been affecting women’s health and quality of life alarmingly [24–26]. In Africa, a large number of women with pelvic organ prolapse and obstetric fistula delayed in seeking medical treatment because of the fear of disclosing the problem due to social stigma [27, 28], and more than 67.7% of women with advanced POP had symptoms of depression and mental dysfunction [29, 30]. The Ethiopian government has yet to design and include POP as a maternal health indicator in the country's demographic and health survey, which will aid in the development of a separate plan to prevent and manage POP in the health sector.

Although rare studies have identified with different study types, still there is a scarcity of locally generated evidence regarding the magnitude and risk factors of POP in Eastern Ethiopia. Therefore, this study aimed to assess the magnitude of POP and associated factors among ever-married women attending healthcare services in public hospitals in Eastern Ethiopia.

## Methods

### Study setting, period, and design

A facility-based cross-sectional study was employed in two public hospitals (Hiwot Fana Specialized University hospital and Jugal regional hospital) found in Harar town, Eastern Ethiopia from March 4th to April 5th, 2020. Harar is a town in Harari regional state, which is located 526 km away from the capital city of Ethiopia, Addis Ababa. According to the 2007 census conducted by the Central Statistics Agency (CSA) in Ethiopia, the total population of the town is 183,415 were 92,316 males and 91,099 females. In the town, there are 47 health facilities (34 health posts, 8 health centers, 5 hospitals, and the Family guidance Association). Among the five hospitals found in the town, only two of them are giving service as public hospitals. This study was conducted in two public hospitals, where different and multidimensional health care services are being provided to the patient [31].

## Population, eligibility criteria, and sampling procedures

All ever-married women attending healthcare services in public hospitals of Harari Regional State were considered as source population. Ever-married women who came for healthcare services at Family planning unit, Adult outpatient Department (OPD), Gynecologic OPD, and Expanded Program of Immunization (EPI) unit were considered for enrollment. Thus, eligible ever-married women attending healthcare services during the data collection period were studied. Women with the following preconditions (pregnant women, critically ill women, and women with mental problems) were excluded from the study. The sample size was calculated using EPI Info version 7 statistical software for the double population proportion formula by considering prolonged labor as an exposure variable [32] with the following assumptions. The proportion of outcomes among unexposed (had no history of prolonged labor) (P = 50.7%), the proportion of outcomes among exposed (had a history of prolonged labor) (P = 64.7%), 95% confidence level, 80% power. Adding 10% contingency for non-response rate, finally, 460 study participants were obtained.

Two public hospitals Jugal Regional Hospital (JRH) and Hiwot Fana Specialized University Hospital (HFSUH)) from Harari Region were selected as the study site. The monthly number of women attending health service in selected units at HFSUH was 400 and JRH was 354. After the monthly average flow number of study participants was identified the total sample size (n = 460) was proportionally allocated to both hospitals. Accordingly, 216 samples were allocated to JRH and 244 were allocated to HFSUH. The study subjects were selected through systematic sampling techniques (i.e. kth value 400/244 = 1.6–2 for HFSUH and 354/216 = 1.6–2 for JRH) every 2nd woman was included in the sample. Finally, the data were collected from eligible participants.

### Data collection tools and procedures

Data were collected using structured interviewer-administered questionnaires. First, the questionnaires were prepared in English language and translated into local languages (Afan Oromo and Amharic) by a bi-lingual expert. Then, they were translated back into the English version to check for consistency. These structured questionnaires have different parts: Socio-demographic characteristics, obstetric and gynecologic history, medical history, and Pelvic Organ Prolapse Simple Screening Inventory questionnaires. The questionnaires were extracted and adopted from different kinds of literature [14, 20]. Data were collected by six female Bachelor of Science nurse professionals who had previous data collection experiences. Two supervisors (BSc public health professionals) were selected to supervise the data collectors and data collection process. The data collectors interviewed the participants after they fully decided to enroll in the study. The interview was conducted in a separate area during the waiting time and exit time to assure privacy. Screening for privacy was assured.

## Study variables and measurement

In this study, the dependent variable was POP. The explanatory variables were: Socio-demographic characteristics (age, religion, ethnicity, marital status, residence, educational status, and occupation). Obstetric and gynecologic history factors (Number of pregnancies (gravidity), history of abortion, number of childbirth (parity), mode of delivery at first childbirth, ever had a vaginal delivery, place of delivery, prolonged labor, time since last birth, family history of POP). Reproductive history-related variables (age at first marriage, history of using family planning, menopause). Medical history-related variables (history of chronic cough, chronic constipation, and maternal obesity).

### Measurements

In this study, the outcome variable (POP) was assessed based on women’s reports of symptoms related to genital prolapse. These symptoms were explored by asking, “Do you have a sensation that there is a bulge in your vagina or that something is falling out from your vagina?” [20], and by asking more questions using the other three Pelvic Organ Prolapse simple screening inventory (POPSSI) [14] questions such as (“Do you experience urinary incontinence with laughing, sneezing or coughing”?), (“Do you experience urinary urgency”?) and, (“Do you feel pain during defecation?”) [14, 33]. Accordingly, if the women responded **“**Yes**”** to the above questions, they were categorized as “have POP” and if their responses were “No”, they were categorized as “have no POP”.

### Data quality control

Before the actual data collection was performed, a pretest of the questionnaire was conducted on 23 samples (5% of the total sample size). The data collectors along with the supervisors were trained for two days regarding the purpose of the study, data collection procedures, and data handling techniques. Close supervision of the data collectors was made on daily basis. The collected data were checked by supervisors and the principal investigator for completeness, and consistency. Double data entry was done by two independent data clerks, and the consistency of the entered data was cross-checked. Simple frequencies were run to check any missing values and outliers and crosschecked with hard copies of the collected data before analysis.

### Data processing and analysis

The collected data were coded, cleaned, and entered into Epi-data version 3.1, and exported to SPSS version 22 (IBM SPSS Statistics, 2013) for further analysis. Descriptive analysis was done using frequency tables, the proportion with 95% CI and summary measures. Bivariable logistic regression analysis was carried out to select candidate variables for multivariable analysis and those variables with a p-value less than 0.25 were considered for the final model of multivariable analysis based on the assumption of selection criteria [34]. The multivariable analysis was performed to identify the true effects of the selected predictor variables on POP. Multi-collinearity test was carried out to see the linear correlation among independent variables by using standard error. Standard error > 2 was considered suggestive of the existence of multi-collinearity. Therefore, variables with standard error > 2 were checked to be dropped. The model adequacy was checked using the Hosmer–Lemeshow goodness of fitness test and the result was found to be insignificant (*p* = 0.677**)** which indicates the model was fitted. Finally, the strength of associations between the outcome variable and predictor variables was assessed using Adjusted Odds Ratio (AOR) with 95% Confidence Intervals (95% CI), and the significance of the association was declared at a p-value of less than 0.05.

## Results

### Socio-demographic characteristics of the study participants

A total of 458 participants were enrolled in the study with a response rate of 99.5%. The mean age of the women was 36.3 years (SD =  ± 14.6) ranging from 15 to 69 years. More than half, 257(56.1%) of the participants were Muslim. Nearly one-fourth of the study participants (22.9%) were not attended formal education and one-fourth of the women, 117(25.5%) were government employees. The majority of women, 301(65.7%) were from urban settings (Table 1).

**Table 1:** Socio-demographic characteristics of the women who attended healthcare services in public hospitals of Harari Regional State, Eastern Ethiopia, 2020 (n = 458)

Characteristics	Categories	Frequency (n)	Percentage (%)
Age (years)	15–24	94	20.5
	25–34	130	28.4
	35–44	87	19.0
	45–54	51	11.1
	= > 55	96	21.0
Ethnicity	Oromo	236	51.5
	Harari	66	14.4
	Amhara	80	17.5
	Tigre	47	10.3
	Other*	29	6.3
Religion	Muslim	257	56.1
	Orthodox	152	33.2
	Protestant	32	7.0
	Other**	17	3.7
Educational status	No formal education	105	22.9
	Can read and write without formal education	85	18.6
	Primary (1–8)	69	15.1
	Secondary (9–12)	101	22.1
	Collage and above	98	21.4
Occupation	House wife	108	23.6
	Government employee	117	25.5
	NGO employee	27	5.9
	Farmer	78	17.0
	Self-employee	84	18.3
	Other***	44	9.6
Residence	Rural	157	34.3
	Urban	301	65.7

Key: * = Gurage, Woliyita, Somali; ** = Catholic, Wakefata, Adventist; *** = Student, daily labror; NGO = Non-governmental Organizations

### Obstetric/gynecologic history-related characteristics

Among 458 ever-married women enrolled in the study, a vast majority, 375 (81.9%) had a history of previous pregnancy, and one-hundred-four (27.7%) of them had a history of abortion, and around 368(98.1%) women had a history of childbirth. More than half, 220 (59.8) of the participants were primiparous. Of 368 women who had a history of childbirth, a vast majority, 326 (88.6%) of them had vaginal delivery for the first childbirth. Nearly half of the study participants (44.0%) had a history of home delivery, and around 88 (23.9%) of them had a history of prolonged labor (Table 2).

**Table 2:** Obstetric and gynecologic history related characteristics of women attending healthcare services in public hospitals of Harari Regional State, Eastern Ethiopia, 2020

Characteristics	Categories	Frequency (n)	Percentage (%)
Ever got pregnant (n = 458)	Yes	375	81.9
	No	83	18.1
Number of pregnancy (gravidity) (n = 375)	1	225	60
	2–4	56	14.9
	> 5	94	25.1
Ever had history of abortion (n = 375)	Yes	104	27.7
	No	271	72.3
Ever had child birth (n = 375)	Yes	368	98.1
	No	7	1.9
Number of child birth (parity) (n = 368)	Primiparous	220	59.8
	Multiparous	148	40.2
Mode of delivery at first child birth (n = 368)	Vaginal delivery	326	88.6
	Caesarean delivery	42	11.4
Ever had vaginal birth (n = 368)	Yes	343	93.2
	No	25	6.8
Ever had caesarean delivery (n = 368)	Yes	89	24.2
	No	279	75.8
Place of delivery (n = 368)	Institutional delivery	206	56.0
	Home delivery	162	44.0
History of prolonged labor (≥ 24 h) (n = 368)	Yes	88	23.9
	No	280	76.1
Time since last birth (n = 368)	6–12 months	109	29.6
	> = 12 months	259	70.4
Family history of pelvic organ prolapse (n = 458)	Yes	40	8.7
	No	418	91.3

### Reproductive health and medical history related characteristics

Of 458 women enrolled in the study, around two-thirds (81.4%) of them were currently married, and more than half, 253 (55.2%) of the women first married at the age of less than 18 years. About 74 (16.2%) of the participants had a history of marrying more than once. Concerning family planning services, more than one-fourth (26.6%) of the women had no history of family planning utilization. Of 458 participants enrolled in the study, around 72 (15.7%) of them had a history of chronic cough, and 40 (8.7%) of them had a history of chronic constipation (Table 3).

**Table 3:** Reproductive health and medical history related characteristics of the women attending healthcare services in public hospitals of Harari Regional State, Eastern Ethiopia, 2020

Characteristics	Categories	Frequency (n)	Percent (%)
Marital status (n = 458)	Currently married	373	81.4
	Divorced/widowed	85	18.6
Age at first marriage (n = 458)	< 18	253	55.2
	> 18	205	44.8
Had history of marriage more than once (n = 458)	Yes	74	16.2
	No	384	83.8
History of family planning utilization (n = 458)	Yes	336	73.4
	No	122	26.6
Menopausal age (n = 458)	Yes	112	24.5
	No	346	75.5
Had history of chronic cough (n = 458)	Yes	72	15.7
	No	386	84.3
Had history of chronic constipation (n = 458)	Yes	40	8.7
	No	418	91.3
Had history of heavy lifting (n = 458)	Yes	74	16.2
	No	384	83.8

### Magnitude of pelvic organ prolapse

Overall, the proportion of Pelvic Organ Prolapse was 48 (10.5%) [95% CI (7.6, 13.5)] based on the women’s reporting of symptoms. Moreover, 48 (10.5%) of them reported that they had felt or seen a bulge in the vagina, 22 (4.8%) had urinary incontinence with laughing, sneezing, or coughing, 26 (5.7%) had urinary urgency and 21 (4.6%) had pain during defecation.

### Factors associated with pelvic organ prolapse

In the final model of multivariable analysis, variables such as women’s age ≥ 55, lifting heavy objects, history of contraceptive utilization, history of chronic cough, and chronic constipation remained statistically significantly associated with POP. Accordingly, the likelihood of POP was 3.5 times higher among women aged greater or equal to 55yeas compared to women whose age was 25 to 34yeas[AOR = 3.51, 95% CI (1.04, 11.76)]. Women who had a history of lifting heavy objects were 3.2 times more likely to encounter POP compared to women who had no history of lifting heavy objects [AOR = 3.22, 95% CI (1.56, 6.67)]. Likewise, the odds of having POP was 2.4 times higher in women who had no history of contraceptive utilization compared to their counterpart (contraceptive users) [AOR = 2.40, 95% CI (1.13, 5.31)]. In addition, the likelihood of encountering POP was 2.5 times higher in women who had a history of chronic cough compared to their counterparts [AOR = 2.51, 95% CI (1.18, 5.31)]. Similarly, the odds of POP were nearly four times higher among women reporting a history of chronic constipation than those who had no chronic constipation [AOR = 3.77, 1.54, 9.22)] (Table 4).

**Table 4:** Multivariable logistic regression analysis of factors associated with POP among ever-married women attended healthcare services in public hospitals of Harari Regional State, Eastern Ethiopia, 2020

Characteristics	Categories	POP		COR (95% CI)	AOR (95% CI)
		Yes (%)	No (%)		
Age (years)	15-24	8 (8.5)	86 (91.5)	1.64 (0.57, 4.68)	1.41 (0.43, 4.61)
	25-34	7 (5.4))	123 (94.6)	1	1
	35-44	12 (13.8)	75 (86.2)	2.81 (1.06, 7.46)	3.16 (0.94, 10.69)
	45-54	7 (13.7)	44 (86.3)	2.80 (0.93, 8.42)	2.91 (1.05, 8.23)*
	>= 55	14 (14.6)	82 (85.4)	3.01 (1.16, 7.75)	3.51 (1.04. 11.76)*
Residence	Urban	24 (8.0)	277 (92.0)	1	1
	Rural	24 (15.3)	133 (84.7)	2.08 (1.14, 3.80)	1.91 (0.97, 3.74)
History of lifting heavy objects	Yes	18 (24.3)	56 (75.7)	3.81 (1.98, 7.26)	3.22 (1.56, 6.67)***
	No	30 (7.8)	354 (92.2)	**1**	1
Family history of Pelvic Organ Prolapse	Yes	8 (20.0)	32 (80.0)	2.36 (1.02, 5.48)	2.14 (0.85, 5.40)
	No	40 (9.6)	378 (90.4)	1	1
History of contraceptive utilization	Yes	29 (8.6)	307 (91.4)	**1**	1
	No	19 (15.6)	103 (84.4)	1.95 (1.05, 3.63)	2.40 (1.13, 5.05)***
History of chronic cough	Yes	15 (20.8)	57 (77.2)	2.82 (1.44, 5.51)	2.51 (1.18, 5.31)***
	No	33 (8.5)	353 (91.5)	1	1
History of chronic constipation	Yes	10 (25.0)	30 (75)	3.33 (1.51, 7.34)	3.77 (1.54, 9.22)**
	No	38 (9.1)	380 (90.9)	1	**1**
Menopausal age	Yes	14 (12.5)	98 (87.5)	1.31 (0.68, 2.54)	0.60 (0.23, 1.58)
	No	34 (9.8)	312 (90.2)	**1**	1

Key: 1= Reference category *= p-value < 0.05, **= p-value < 0.001, ***= p-value < 0.001, COR = crude odds ratio, 495 AOR = adjusted odds ratio

## Discussion

In this study, the overall magnitude of POP was 10.5%. In other words, one in ten ever-married women who visited health facilities for various reasons has pelvic organ prolapse in Harari regional state, Eastern Ethiopia. This current proportion of POP is nearly comparable to previous research reports from Eastern Ethiopia (9.5%) [20], Benchi Maji Zone of Southern Ethiopia (13.3%) [31], Ghana (12.1%) [35], Tanzania (14.4%) [36], China (9.10%) [37], and North Carolina (12.6%) [18]. However, the proportion of Pelvic Organ Prolapse reported in this study was higher compared to previous studies done in different parts of the world like 6.3% in Gondar, Northwest Ethiopia [32], 3.6% in France [38], and 5.4% in Nigeria [39], and 1.4% in Sokoto [9]. The possible justification for this discrepancy might be due to the socio-demographic characteristics of the study populations. In Eastern Ethiopia, women are more engaged in heavy workloads such as carrying Khat and other cash crops to the market because of the socio-economic and cultural characteristics of the Eastern part of the country. Moreover, health service accessibility and exposure to information regarding healthcare services might be lower in the former study population because the majority of current study participants were from marginalized rural women of Eastern Ethiopia.

In contrast, the result of this study is encouraging as the current prevalence of POP is comparatively lower than the previous studies reports in Ethiopia (46.7%) [40], Arab United Emirates(29.6%) [41], South Africa(57%) [26], and Tanzania (64.4%) [6]. The possible justification for these differences can be explained by the time gap between study periods, geographical setting of the study population, and difference in the sample size of the study. Furthermore, the discrepancy in estimates could be due to the tool used to classify women with POP. Home-based questionnaire interviews were used in the Dabat district of Ethiopia and Tanzania, and the women were then invited to the local health clinic for a pelvic examination. Another possible explanation might be because currently the government is increasing the number of health extension workers in the rural community and introducing a community health insurance program, which is motivating communities toward health services utilization, and the women accessed the healthcare before the problem is getting worse.

In the final model of multivariable analysis, the odds of POP were more than three times higher among women aged 55 and above compared to women aged 25 to 34yeas. This is comparable with the finding of a previous study conducted in Baherdar, Ethiopia [42], Uganda [43], and Nepal [44]. The justification for this is because the risks of Pelvic Organ Prolapse increase with advanced maternal age and, this is because as women age getting advance, the pelvic floor muscles become weak to support pelvic organs, which may lead to uterovaginal prolapse. However, this result is inconsistent with study conducted in Bench Maji Zone in which age group 31–40 years and 41–50 years were also risk for pelvic organ prolapse [31]. This inconsistence might because of socio-economic factors of the study participant such as nutritional status.

In this study, lifting heavy objectives was found to be an independent predictor of POP. Women who had a history of lifting heavy objects were 3.2 times more likely to encounter pelvic organ prolapse than those with no history of lifting heavy loads. This is supported by studies done in Dabat district [32] and Bahir-dar [42], Ethiopia. It is also consistent with previous studies' reports from Tanzania [6], Australia [36], and Addis Ababa, Ethiopia [14]. The possible explanation for this might be due to the fact that lifting heavy object may increase intra-abdominal pressure and causes damage to the pelvic floor muscles. This can weaken the muscles responsible for supporting the pelvic organs because of pressure effect from hanging down [45]. In addition, pelvic floor muscles that support the pelvic organs become stretched, damaged or weakened, causing the organs they support to drop downward.

In addition, a history of chronic cough was another factor independently associated with POP. The odds of POP was 2.5 times higher in women with a history of chronic cough compared to their counterpart. This result is in line with studies conducted in Jimma Ethiopia [22], Nigeria [10], United Arab Emirates [41], and China [37]. The possible explanation could be justified by women who have chronic cough are at risk of genital prolapse because of a long-term effect of chronic cough that increases intra-abdominal pressure. Likewise, having a history of chronic constipation is also independently associated with POP. The odds of pelvic organ prolapse were nearly four times higher among women reporting a history of chronic constipation than those who had no chronic constipation. This finding is also supported by studies conducted by Forner et al., Australia [36], Akmel et al., Ethiopia [22], Elege et al., Nigeria [10], and Li et al., China [37]. This is because chronic straining with bowel movements due to constipation can increase the risk of uterovaginal prolapse.

Furthermore, in this study, women who had no history of contraceptive utilization were more likely to encounter Pelvic Organ Prolapse compared to those who utilized the services. The possible justification for this might be because using contraception can reduce short birth intervals and multiple childbirth, which may increase the risks of POP.

Furthermore, although we did not report in the final model of multivariable analysis, multiparity and prolonged labor were factors independently associated with POP in bivariable analysis. These are also reported in a multivariable analysis of previous literature conducted elsewhere [9, 40, 46]. This might be because labor by itself can cause damage to the pelvic floor muscle, especially during the second stage of labor when the fetal head places the pelvic floor muscles under considerable stretch and when it is prolonged, the damage is the extent [45]. Similarly, with multiple vaginal deliveries, the muscles and ligaments around the uterus can be weakened, so that they can no longer support the weight of the uterus.

### Strengths and limitations

Using validated data collection tools is the strength of this study. Limitations: Since the cross-sectional survey, the real causal association could have not been determined. The study was conducted only among women who visited public hospitals; women who attended private health facilities were not enrolled. Also, women who had undergone previous POP surgery were part of the study. Since we have not done pelvic examinations, the classification of types of pelvic organ prolapse was ascertained.

## Conclusion

The study revealed that one in ten ever-married women who visited health facilities for various reasons have pelvic organ prolapse in Harari regional state, Eastern Ethiopia. This finding shows how a staggering number of women suffer from pelvic organ prolapse. Women’s age greater than and equal to 55 years, history of lifting heavy objects, and having a history of chronic cough and chronic constipation were independent predictors of POP. Therefore, efforts are needed to emphasize the prevention and treatment of chronic medical conditions like chronic cough and chronic constipation. Healthcare providers should give due attention to women exposed to heavy workloads especially those of advanced age. Further studies with pelvic examination using POP-Q staging to know the true effects of contributing factors to POP are also needed.

## Data Availability

The data set generated or analyzed during the current study are not publicly available due to the privacy of the participants and institution restriction but are available from the corresponding author upon reasonable request.

## Abbreviations

ACOG: American College of Obstetricians and Gynecologists
CSA: Central Statistics Agency
POP: Pelvic Organ Prolapse
HFSUH: Hiwot Fana Specialized University Hospital
ICS: International Continence Society
POP-Q: Pelvic organ prolapse-quantification
POPSSI: Pelvic organ prolapse simple screening inventory
SPSS: Statistical Package for Social Science
WHO: World Health Organization
